# A novel strain of acetic acid bacteria *Gluconobacter oxydans* FBFS97 involved in riboflavin production

**DOI:** 10.1038/s41598-020-70404-4

**Published:** 2020-08-11

**Authors:** Abeer Essam Noman, Naif S. Al-Barha, Abdul-Aziz M. Sharaf, Qais Ali Al-Maqtari, Amani Mohedein, Hammad Hamed Hammad Mohammed, Fusheng Chen

**Affiliations:** 1 Hubei International Scientific and Technological Cooperation Base of Traditional Fermented Foods, College of Food Science and Technology, Huazhong Agricultural University, Wuhan, 430070 China; 2 Department of Food Science and Technology, Faculty of Agriculture, Sana’a University, Alwehdah street, P. O. Box 19509, Sana’a, Yemen; 3 School of Physical Education and Sport Training, Shanghai University of Sport, Shanghai, China; 4 Department of Physical Activity, Faculty of Education and Science, Rada’a, Albaydha University, Albaydha, Yemen; 5 Faculty of Communication, Media and Broadcasting, Limkokwing University of Creative Technology, Cyberjaya, Malaysia; 6 State Key Laboratory of Food Science and Technology, Jiangnan University, Wuxi, 214122 Jiangsu China; 7 College of Food Science and Technology, Huazhong Agricultural University, Wuhan, 430070 China; 8 Ministry of Agriculture and Forestry, National Food Research Center, P. O. Box 213, Khartoum, Sudan

**Keywords:** Biotechnology, Industrial microbiology

## Abstract

A novel bacterial strain of acetic acid bacteria capable of producing riboflavin was isolated from the soil sample collected in Wuhan, China. The isolated strain was identified as *Gluconobacter oxydan*s FBFS97 based on several phenotype characteristics, biochemicals tests, and 16S rRNA gene sequence conducted. Furthermore, the complete genome sequencing of the isolated strain has showed that it contains a complete operon for the biosynthesis of riboflavin. In order to obtain the maximum concentration of riboflavin production, *Gluconobacter oxydans* FBFS97 was optimized in shake flask cultures through response surface methodology employing Plackett–Burman design (PBD), and Central composite design (CCD). The results of the pre-experiments displayed that fructose and tryptone were found to be the most suitable sources of carbon and nitrogen for riboflavin production. Then, PBD was conducted for initial screening of eleven minerals (FeSO_4_, FeCl_3_, KH_2_PO_4_, K_2_HPO_4_, MgSO_4_, ZnSO_4_, NaCl, CaCl_2_, KCl, ZnCl_2_, and AlCl_3_.6H_2_O) for their significances on riboflavin production by *Gluconobacter oxydans* strain FBFS97. The most significant variables affecting on riboflavin production are K_2_HPO_4_ and CaCl_2_, the interaction affects and levels of these variables were optimized by CCD. After optimization of the medium compositions for riboflavin production were determined as follows: fructose 25 g/L, tryptone 12.5 g/L, K_2_HPO_4_ 9 g/L, and CaCl_2_ 0.06 g/L with maximum riboflavin production 23.24 mg/L.

## Introduction

Riboflavin or the so-called vitamin B2 is a water-soluble vitamin that belongs to the B vitamins complex group, a vital component of the energy metabolism, and a bioactive molecule that has an important role in various cellular functions^1,2^. In addition, to its role as the precursor of flavin adenine dinucleotide (FAD) and flavin mononucleotide (FMN), the major products of riboflavin that act a fundamental role in metabolism, acting as cofactors for a wide variety of enzymes intermediating many redox reactions in the cell^3,4^. In contrast, to many micro-organisms and plants, humans and animals cannot synthesize this vitamin, and thus need to supply their diet with external riboflavin to meet their nutritional requirements^5^. Riboflavin is used on a large scale as food and feed additives, food colorant, and pharmaceutical preparations. The commercial production of this vitamin can be accomplished by chemical synthesis or biological synthesis, yet in recent times the chemical synthesis has totally replaced to the microbial fermentation because of its cost-effectiveness, reduction in waste and energy requirements, and the use of renewable resources^6^. At present, several species of bacteria and fungi are harnessed for industrial riboflavin production. The bacterium *Bacillus subtilis* and the fungus *Ashbya gossypii*, as well as the yeast *Candida famata* are mainly industrial strains for riboflavin production^7^. Furthermore, other species such as *Candida ammoniagenes*, *Pichia gulliermondii*, *E. coli*, and *Eremothecium ashbyii* are engineered for the production of riboflavin^8,9^. The biosynthesis pathway of riboflavin production has been studied in both Gram-negative and Gram-positive bacteria, but it has been studied widely in two species namely *Escherichia coli* and *Bacillus subtilis*^10^*.* Acetic acid bacteria (AAB) are distinguished as Gram-negative obligate aerobic bacteria belonging to the family *Acetobacteraceae* mostly isolated from plants, flowers and fruits as well as from fermented foods, and beverages^11,12^. AAB are considered an important type group of bacteria involved in the production of several compounds used in chemical, pharmaceutical, medical, and biotechnological fields mainly due to their high ability to partially oxidize a variety range of alcohols, carbohydrates, and sugar alcohols. As a result of this process, large amounts of industrial interesting products are released and accumulated directly in the culture media^13,14^,. This type of metabolism is known as “oxidative fermentation” that distinguishes AAB from other types of bacteria and allows them to play an important role as biocatalysts for the progress of eco-friendly bio-based processes as an alternative to the artificial processes^15,16^. AAB species perform many roles in biotechnology applications, but strains of *Gluconobacter* genus are particularly industrial important, where they are already exploited for the synthesis of vitamin C (ascorbic acid), the antidiabetic drug Miglitol, and to improve the efficiency of 5-ketofructose formation from fructose^17–19^. They are also used in the production of erythrulose and dihydroxyacetone (DHA) which are mostly utilized as tanning agents in the cosmetic industry and as a precursor for the pharmaceuticals and chemicals industry^20,21^. Another important property of AAB is their capability to synthesis different types of exopolysaccharides (EPS) such as levan, detran, acetan, and cellulose, the latter is the most valuable compound produced by species of the *Komagataeibacter* and *Gluconacetobacter* genera with unique features. Moreover, AAB are involved in the production of several kinds of fermented foods and beverages such as vinegar, kombucha tea, beer, and kefir^22–26^. The purpose of this work was to characterize a new bacterial strain FBFS97 of acetic acid bacteria, which was identified based on its 16S rRNA sequence, phenotype and biochemical characteristics, and to optimize its fermentation composition media through statistical experimental design method to enhance its efficiency for riboflavin production, which could, in turn, lead to the discovery of a new potential resource of riboflavin production. Up to now, no study at all has been carried out to investigate the production of riboflavin by acetic acid bacteria.

## Material and methods

### Strain, media and culture conditions

An acetic acid bacteria strain used in this study were isolated from soil samples collected from different locations of Wuhan, China. GYC medium comprised of glucose 60 g, yeast extract 10 g, CaCO_3_ 30 g, Agar 15 g, and 1000 mL distilled water, after that the plates were incubated at 28 °C for 3 days. The isolated bacterial strain maintained as frozen stock in GY broth (glucose 10 g, yeast extract 1 g, and 100 ml distilled water) containing 20% (v/v) glycerol at − 80 °C for consequent investigations.

### Morphological, biochemical, and physiological characteristics

The isolated strain FBFS97 was examined for morphological properties according to the methods described previously^27^. The ability to produce 2-keto-D-gulonate, 2,5-Diketo-d-gluconate, and 5-keto-D-gulonate was performed through detection using the HPLC technique as reported before by Blake et al.^28^. Catalase and oxidase activities, utilization of different carbon sources, acetic acid production, as well as the isolated bacterial strain growth at different temperatures and pH values were investigated as reported by Yamada and others^29–31^.

### Genome sequencing and assembly

Total genomic DNA of FBFS97 was extracted using the GenElute Bacterial Genomic DNA kit (Sigma) according to the manufacturer’s recommendations. FBFS97 whole genome was sequenced using the PacBio RSII platform utilizing 2 single- molecule real-time (SMRT) cells, was used to acquire the raw sequence reads at the Genome Technology Facility (GTF) in Lausanne, Switzerland. The high- quality reads were then assembled de novo into a single contig using Hierarchical Genome Assembly Process version 3.0 (HGAP 3.0) in SMRT Analysis version 2.3.0. Automatic annotation and gene prediction were carried out using Prokka version 1.1.0. Circos was used to create the genome circle (https://circos.ca/tutorials/lessons/).

### Molecular identification by 16S rRNA and phylogenetic analysis

The 16S rRNA gene fragment was amplified using universal primers (27F 5′-AGAGTTTGATCMTGGCTCAG-3′ and 1492R 5′-TACGGYTACCTTGTTACGACTT-3′). The amplification profile included an initial denaturation at 94 °C for 4 min, followed by 30 amplification cycles of 94 °C for 30 s, 55 °C for 30 s and 72 °C for 1.5 min. The PCR product was sent to Shanghai Sangon Biological Engineering Technology & Services Co., Ltd., for sequence determination. The phylogenetic tree was constructed through the neighbor joining (NJ) method of MEGA 7.0 software^32^.

### Chromatography conditions

A high-resolution UHPLC Dionex Ultimate 3,000 coupled with a Q-Exactive hybrid quadrupole-orbitrap mass spectrometer was applied. In brief, 5 ml of culture suspension was centrifuged and filtrated with a 0.22 µm membrane. 20 µl of the filtrated sample was injected into an Accucore aQ C18 Polar Endcapped LC column (150 mm × 2.1 mm, 2.6 µm) at 25 °C and the flow rate was 0.2 mL/min. The analysis was achieved at 270 nm with 30% (v/v) methanol, 70% (v/v) water and 0.1% (v/v) formic acid as a mobile phase. The detection of riboflavin was made in positive electrospray ionization mode (ESI +). Riboflavin was identified based on the retention time and fragmentation pattern of a known standard.

### Riboflavin production conditions

The fermentative production of riboflavin by FBFS97 was carried out in 250 mL Erlenmeyer flasks containing 50 mL of fermentation media (according to the experiment). The inoculated flasks were incubated at 28 °C on a rotatory incubator shaker at 180 rpm for 72 h in aerobic conditions. After incubation time, the samples were collected by centrifugation at 8,000 rpm for 5 min. A 0.8 mL of the culture supernatant was mixed with 0.2 mL of 1 M NaOH, and then 0.4 ml of the resulting solution was neutralized with 1 mL of 0.1 M potassium phosphate buffer (pH 6.0). The concentration of riboflavin was determined by measuring the absorbance at 444 nm. The standard curve was constructed using pure riboflavin standard (Sigma).

### Experimental designs and statistical analysis for medium optimization

Optimization of the fermentation medium was carried out using PBD followed by CCD.

### Screening of the main minerals influencing on riboflavin production using PBD

To study the significant variables for riboflavin production, eleven selected minerals were screened out using Plackett–Burman design by Minitab 18. The PB experiment was carried out in 12 runs according to PB is K + 1 where K indicates to the number of variables, each variable was represented at two levels, maximum and minimum which are denoted by ( +) and (-), respectively. The levels of each variable are listed in (Table 6). All experiments were accomplished in triplicate and the average of riboflavin production was treated as response. The model was subjected to the analysis of variance (ANOVA). *P*-value was used to calculate the significance of the factors where, factors with a significance (P < 0.05) were considered to have a significant effect on riboflavin production. Plackett–Burman experimental design is based on a first order model:

1 $$Y =\upbeta _{0} + \sum {\upbeta {\text{X}}_{{\text{i}}} }$$

where Y is the response or dependent variable (riboflavin production), β_0_ is the model intercept, β_i_ is the regression coefficient, and X_i_ is the level of the independent variable.

### Central composite design (CCD)

In this study, four significant minerals which exerted a positive effect on riboflavin production were used to optimize by employing CCD, each at five coded levels, very low level, low level, central level, high level and very high level indicated as − 2, − 1, 0, 1, 2 respectively. The CCD experiments consisted of 30 trails were designed using Design-Expert 12 (StatEase, Inc., USA). All experiments were done in duplicate and the average of riboflavin production obtained was taken as the response. The production of riboflavin was analyzed with statistical software package Design-Expert 12. After the responses were measured for each trail, each trail was fitted to an independent second order polynomial equation:

2 $$Y =\upbeta _{0} + \sum {\upbeta _{{\text{i}}} {\text{X}}_{{\text{i}}} } { + }\sum {\upbeta _{{{\text{ii}}}} {\text{X}}_{{\text{i}}}^{2} } + \sum {\upbeta _{{{\text{ij}}}} {\text{X}}_{{\text{i}}} {\text{X}}}$$

### Model validation experiment

In order to estimate the accuracy of the optimum medium composition which predicted by the response surface model. All validation experiments have been performed using the medium composition resulting from the model in triplicate.

## Results and discussion

### Morphological, cultural and physiological characterization

The isolated strain FBFS97 exhibited the general morphological, cultural and physiological characteristics of the AAB as was shown in (Table 1). FBFS97 cells were red shaped and Gram-negative. It was also a strictly aerobic, non-spore forming, motile strain, as well as catalase-positive, and oxidase-negative . Colonies of this strain were beige, circular, smooth, and raised to convex with an entire margin, glistening, and formed transparent zones through using CaCO_3_ to produce acetic acid on GYC agar plates. It can produce acetic acid from d-glucose and ethanol but did not able to overoxidise acetic acid to CO_2_ and H_2_O.Also, d-gluconic acid, 2,5-diketo-gluconic acid and a water-soluble brown pigment were produced from d-glucose. FBFS97 exhibited glucose tolerance up to 10% (w/v). It was able to grow at pH 3 and 37 °C but the optimum growth temperature was between 25 and 30 °C and optimal pH was 6. The results of acid production with different sugars displayed that FBFS97 produced acid from sucrose, D-mannitol, D- glucose, maltose, D-galactose, D-sorbitol, and D- fructose but not from α-lactose, β-cyclodextrixs, and lanolin (Tables 1,2).

**Table 1:** Morphological, physiological and biochemical properties of the isolated strain FBFS97.

Parameters	FBFS97
**Morphological characteristics**	
Cell shape	rod
Gram stain	−
Motility	+
Spore	−
**Biochemical characteristics**	
Catalase	+
Oxidase	−
KOH	+
Production of brown pigment on (GYC)	+
Production of acetic acid from ethanol	+
2,5-Diketo-d-gluconate formation	+
Production of acetic acid on GYC agar	+
2-keto-D-gulonate	+
Oxidation of acetic acid to CO_2_ and H_2_O	−
**Utilization of different carbon sources**	
α-Lactose	−
D-Mannitol	w
Sucrose	+
d-Glucose	+
β-Cyclodextrixs	−
Lanolin	−
Maltose	vw
D-Galactose	+
D-Sorbitol	+
D-Fructose	+
**Physiological characteristics**	
Growth at 20 °C	w
Growth at 25 °C	+
Growth at 30 °C	+
Growth at 35 °C	+
Growth 37 °C	vw
pH 4	+
pH 7	+
Growth on nutrient agar	−
Tolerate up to 10% glucose	+

+ Positive, − negative, *w* weakly positive, *vw* very weakly positive.

**Table 2:** Differential characteristics of isolated strain FBFS97 and its phylogenetically nearest relatives in the genus *Gluconobacter*.

Characteristics	97	Go	Gr	Gk	Gz
Water- soluble brown pigment	+	− ^b^	−	+	+
2,5-diketo-d-gluconate	+	− ^b^	−	+	+
Growth at 37 °C	+	+	−	−	+
Acid production from: Sucrose D-sorbitol Maltose Mannitol	+	−	+	− ^b^	−
	+	+	+		+
	vw	w	−	nd	−
	+	w	w	−	+
DNA G + C (mol %)	66.6	60.3	60.5	59.5	60.5

97, FBFS97; Go, *G.oxydans* NBRC14819^T^; Gr, *G. roseus* NBRC3990^T^; Gk, *G. kanchanaburiensis* LMG 26774^ T^; Gz, *G.* ZW160-2^ T^. + , Positive; − , negative; b, some strains in the genus are positive; vw, very weakly positive; w, weakly positive;nd, not determined.

Data cited from Spitaels and others^33,34^.

### Phylogenetic analysis

**Figure 1:**
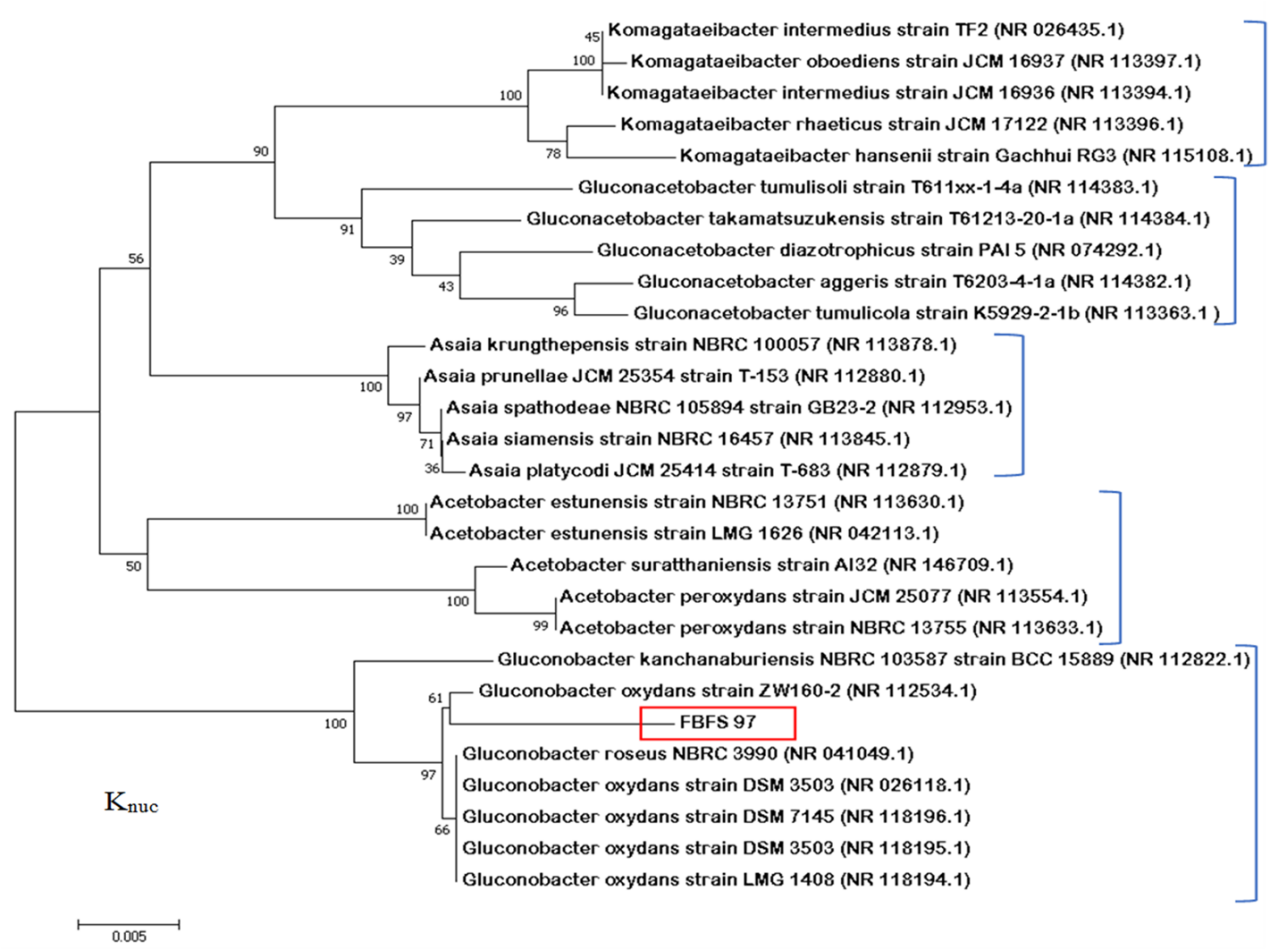
Molecular phylogenetic analysis by Neighbour-joining method based on 16S rRNA gene sequences. The tree is shown the relationship between FBFS97 strain and related species of the genus Komagataeibacter, Gluconacetobacter, Asaia, Acetobacter, and Gluconobacter. Only booststrap values higher than ≥ 50 % expressed as percentages of 500 replications, are shown next to the branch points. The accession numbers of the GenBank database sequence are indicated in parentheses after the strain names, and the newly isolated strain is boxed in red. The branch lengh was expressed in 0.005 unit.

To ascertain the phylogenetic positions of FBFS97 in *Acetobacteraceae*, a phylogenetic tree was generated by the comparison of 16S rRNA gene sequences of FBFS97 strain with those other Acetobacteraceae retrieved from GenBank database according to the bootstrap test of neighbor-joining method of Saitou and Nei^35^ with MEGA 7.0^32^. The phylogenetic tree analysis clearly revealed that the strain FBFS97 was clustered with the type strain *Gluconobacter oxydans* strain ZW160-2 (Accession No. NR_112534.1, similarity 98.41%), and was closed in a single clade together with *Gluconobacter oxydans* strain DSM 7145 (Accession No.NR_118196.1, similarity 98.07%),*Gluconobacter oxydans* strain LMG 1408 (Accession No.NR_118194.1, similarity 98.07%), *Gluconobacter oxydans* strain DSM 3503 (Accession No.NR_118195.1, similarity 97.89%), *Gluconobacter roseus* NBRC 3990 (Accession No.NR_041049.1, similarity 98.07%),and *Gluconobacter oxydans* strain DSM 3503 (Accession No.NR_026118.1, similarity 98.07%). Based on the previously collected data of the morpological, biochemical and physiological characteristics of the isolated strain FBFS97, this strain is most closely to its closest phylogenetic neighbour *Gluconobacter oxydans* strain ZW160-2 (Accession No. NR_112534.1). Thus, FBFS97 strain could be confidently attributed to this species, and was identified as *Gluconobacter oxydans* strain FBFS97 (Fig. 1).

### Genome features and identification of riboflavin in the * G. oxydans* FBFS97 fermentation media

**Figure 2:**
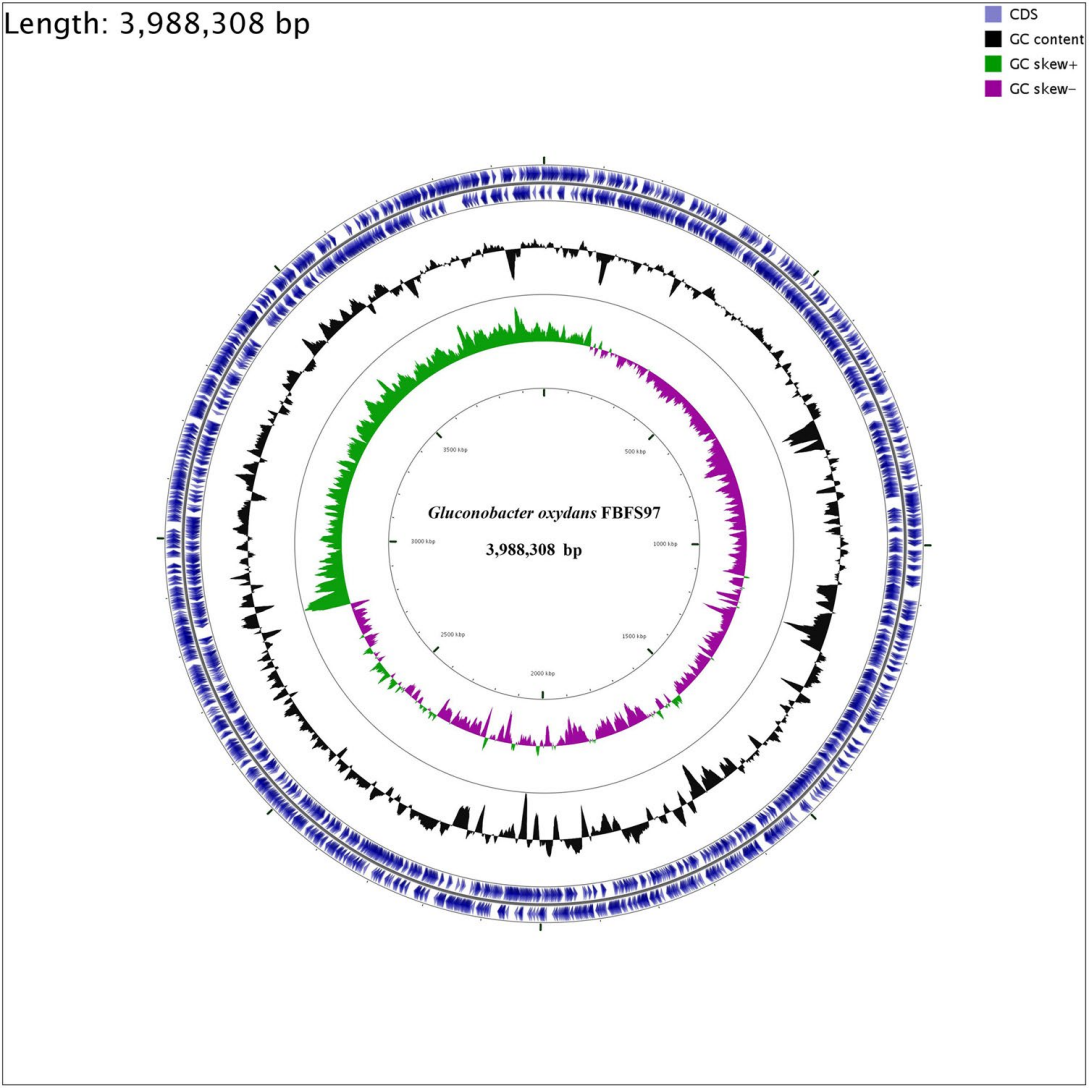
Circle map of the *G.oxydans* FBFS97 chromosome. The inner circle represents GC skew; the middle circle represents G+C content, and the last circle indicates percent genes.

**Figure 3:**
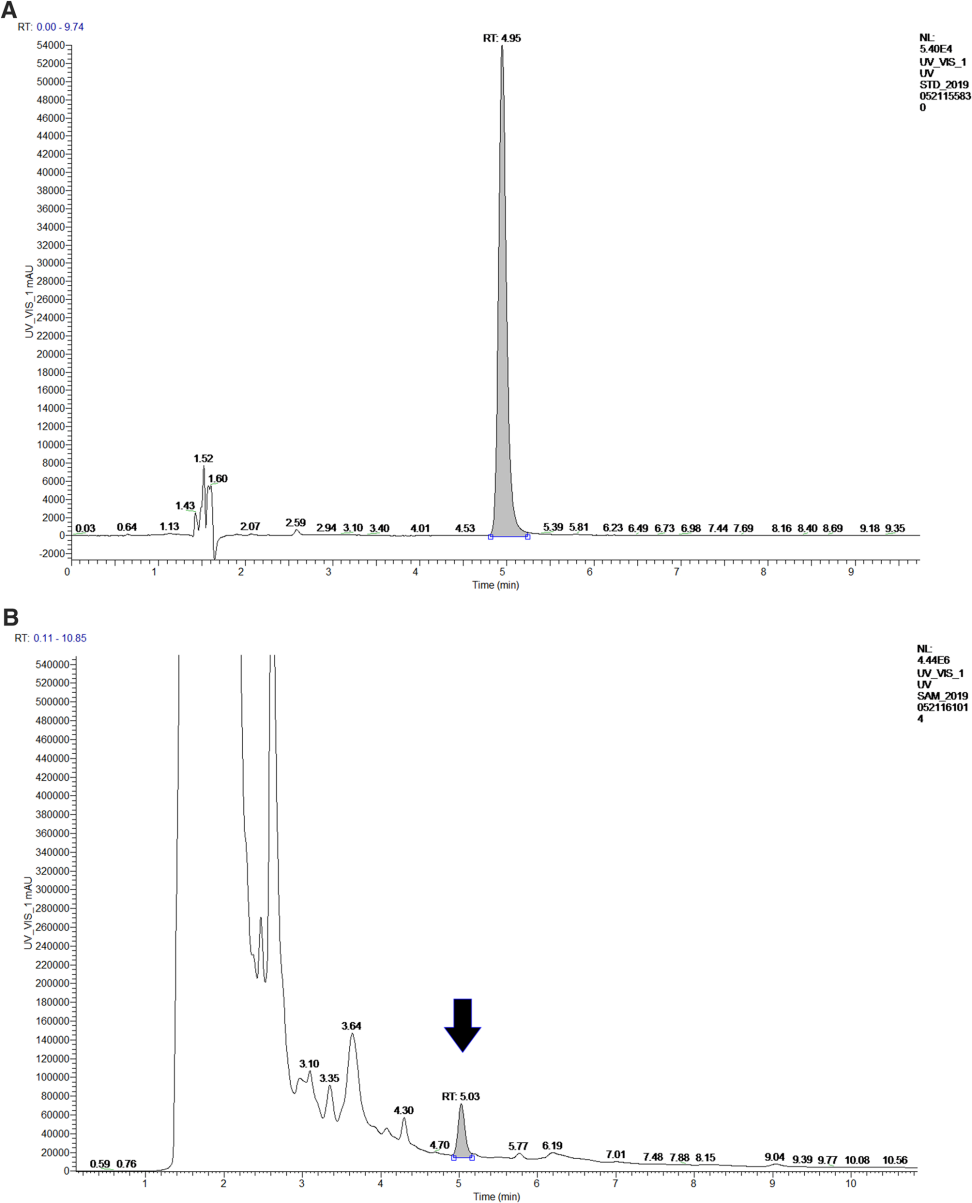

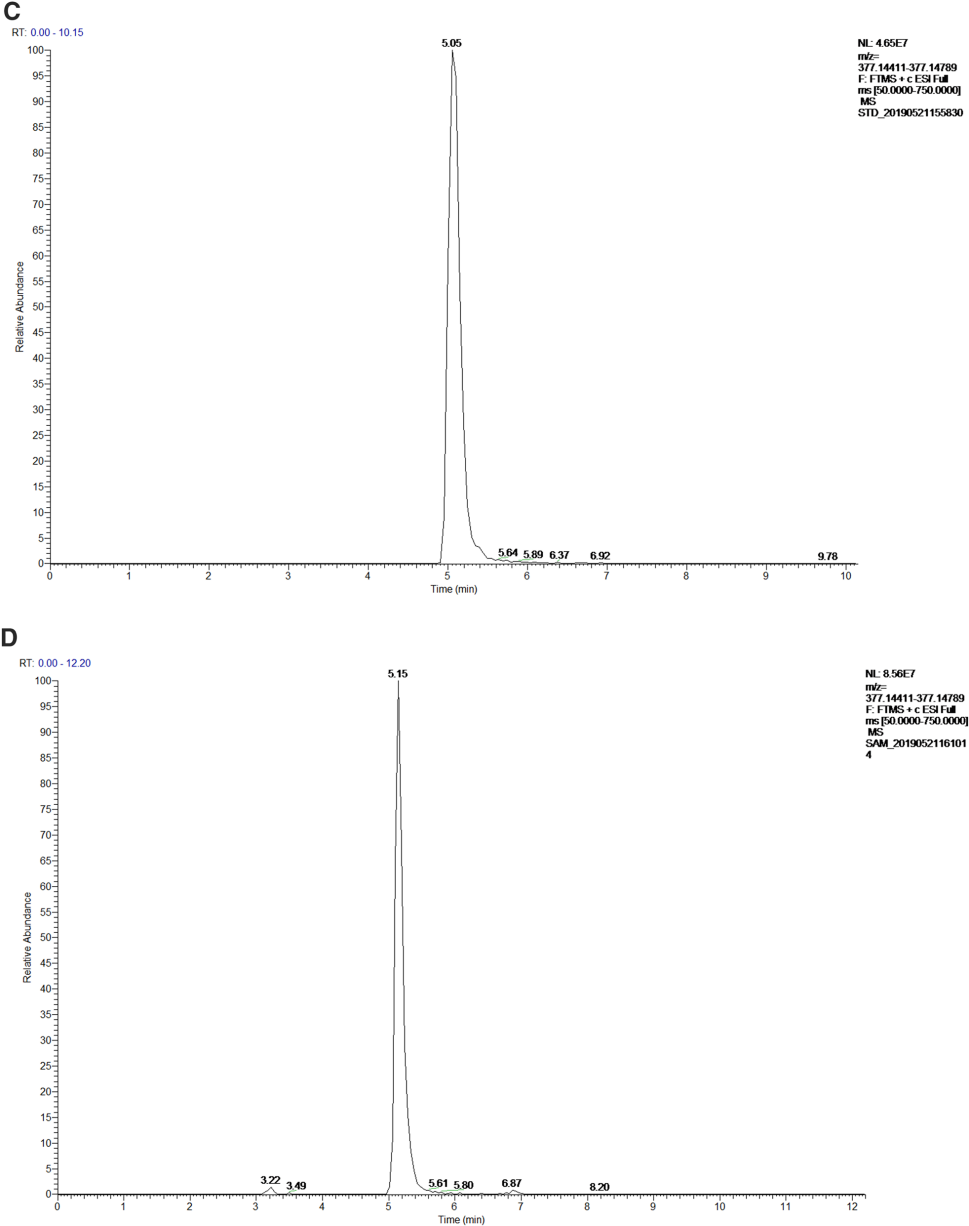

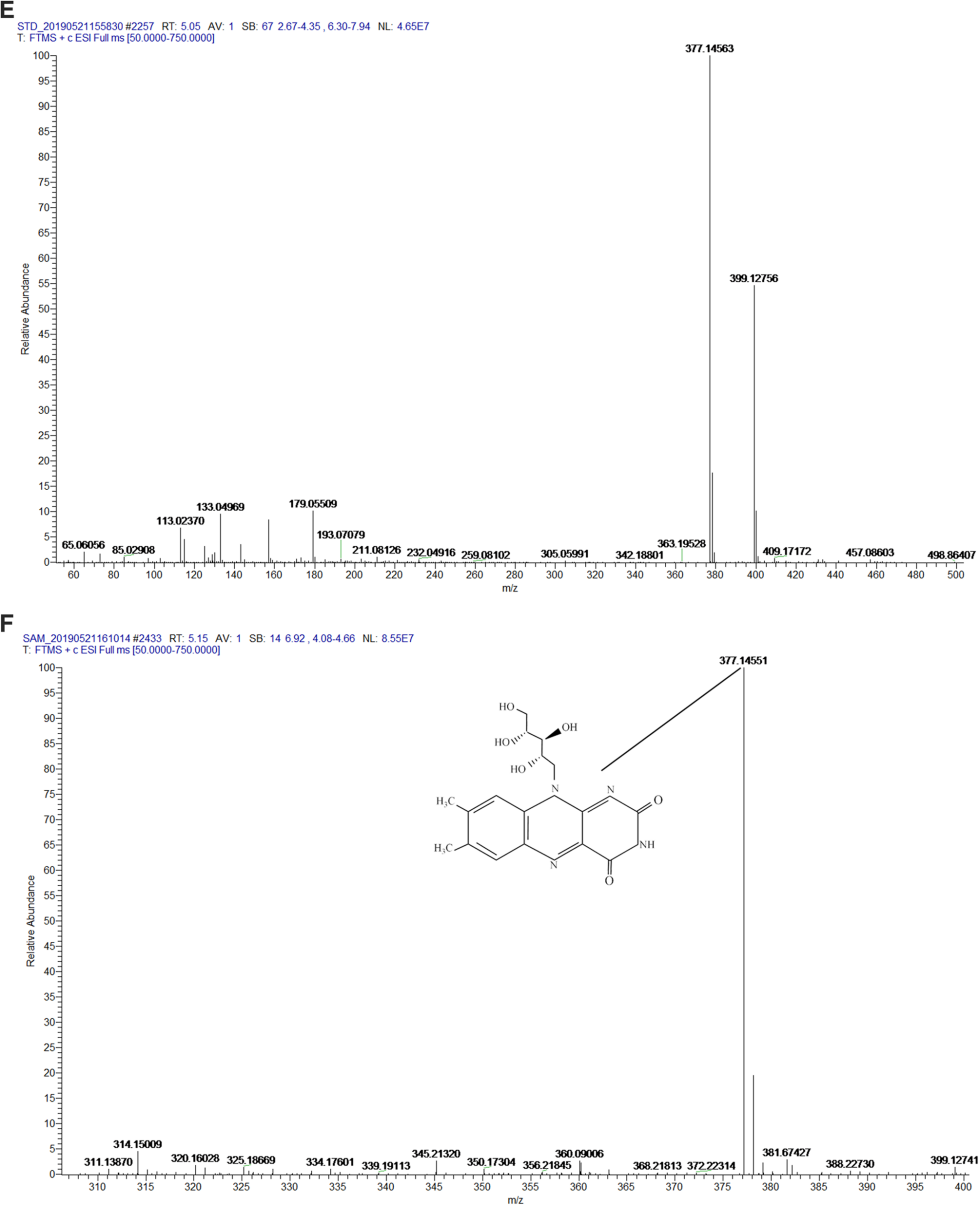
Identification of riboflavin by UHPLC-Q -Exactive. Riboflavin standard UV-VIS spectrum (A), UV-VIS spectrum of the fermentation broth of FBFS97 (B), ion chromatogram of riboflavin standard (C), total ion chromatograms of the identified riboflavin peak in the fermentation broth of FBFS97 (D), ESI-MS/MS spectrum of the riboflavin standard (E) in the positive ion mode, ESI-MS/MS spectrum of the identified riboflavin peak in FBFS97 fermentation broth (F) in the positive ion mode.

To investigate the riboflavin production related genes, the genome of *Gluconobacter oxydans* FBFS97 was sequenced, annotated and explored. The complete genome of FBFS97 is comprised of a single circular chromosome of 3,988,308 bp with an average coverage of 245 x, and a G + C content of 66.6% (Fig. 2). The prediction and annotation of FBFS97 genome resulted in 3,582 ORFs ,and 76.83% of the genome were assigned to the genes with predicted function. Also, the genome contains 12 ribosomal RNA operons (5S, 16S, 23S) , 59 tRNA and 11 other RNA genes. The complete genome of FBFS97 has a total of 3,500 putative coding sequences, among which 2,752 are assigned a putative function, and 748 encode hypothetical proteins. The genome properties and statistics are summarized in Table 3. Based on the Uniprot alignment^36^ and KEGG database^37^ in FBFS97 genome, the full set of riboflavin production related genes were annotated. The putative genes encoding of the riboflavin synthesis pathway on the genome of this strain is represented by RibBA, RibD1, RibD2, PYRP2, Rib4, Rib5, RibF, and bluB. In order to confirm the production of riboflavin in the fermentation broth of FBFS97, UHPLC-MS/MS was used. Riboflavin was successfully detected and identified with the reference standard utilizing the positive ion mode. The MS/MS analysis of the identified riboflavin peak in the FBFS97 fermentation broth was shown a [M + H]^+^ ion at *m/z* 377.14551, which displayed the same [M + H]^+^ ion of the riboflavin standard at *m/z* 377.14563 (Fig. 3).

**Table 3:** General features of the *G.oxydans* FBFS97 genome.

Feature	Value
Genome size	3,988,308 bp
Contigs	1
G + C content (%)	66.6%
Coding sequences (CDSs)	3,500
No. of RNA genes	82
rRNAs (5S, 16S, 23S)	12(4,4,4)
tRNAs	59
Other RNA genes	11
Genes with function prediction	2,752
Genes without function prediction	748

### FBFS97 growth and riboflavin production

**Figure 4:**
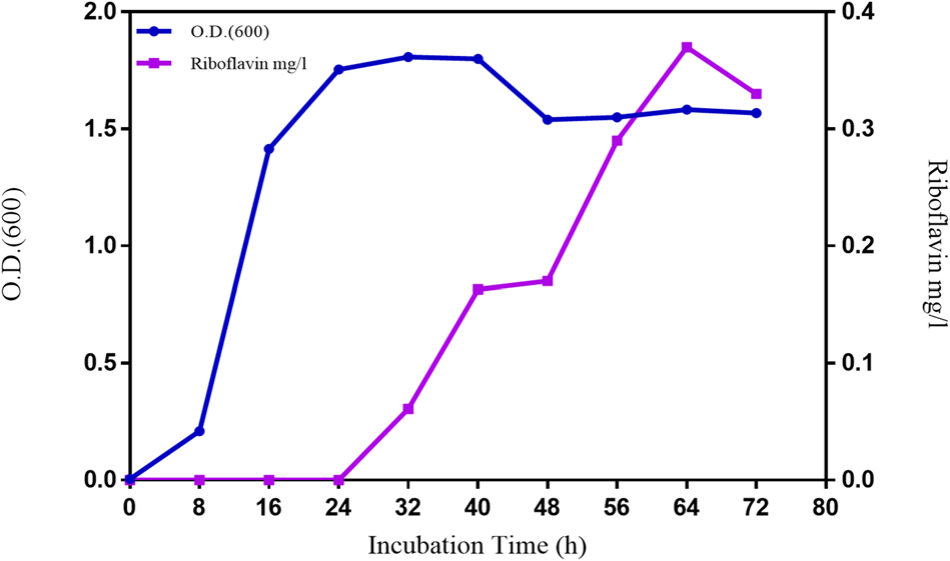
Growth and riboflavin production of *G.oxydans *FBFS97 in fermentation media, the results represent mean values for three shake flasks.

The production of riboflavin by *G.oxydans* FBFS97 was detected at different time points over the range of 0 – 72 h. The growth started in the first hours of incubation, however, a slight riboflavin production was initiated at about 24 h. The production increased markedly at about 32 h when the growth was almost finished. The maximum riboflavin production was 0.33 mg/L at about 64 h, after this point, the production of riboflavin was started to decrease continuously until the end of the incubation period (Fig. 4). The medium pH quickly dropped from pH 6.0 on the first day to pH 2.0 at the end of incubation, which had a negative effect on riboflavin production. At pH 2.0 the riboflavin production starts to decrease until has become negligible or absent after 72 h of the incubation time^38^.

### Screening of the main minerals affecting on riboflavin production using  PBD

Pre-experiments were carried out to investigate the effect of four various carbon sources (glucose, fructose, maltose, and mannitol) and four different nitrogen sources (yeast extract, tryptone, peptone, and malt extract) on riboflavin production by *G.oxydans* FBFS97. The results obtained were analyzed by using a one way ANOVA test. The highest level of riboflavin was observed in the presence of fructose and tryptone (Tables 4, 5). Hence, fructose and tryptone were used as carbon and nitrogen sources for further experiments. The results showed that this improved medium was capable of enhancing riboflavin production from 0.33 mg/l to more than 5 mg/L in 64 h.

**Table 4:** The effect of different carbon sources on riboflavin production by *G.oxydans* FBFS97.

Carbon sources	Riboflavin production mg/L
Glucose	0.033 ± 0.01
Fructose	5.31 ± 0.33
Maltose	1.2 ± 0.05
Mannitol	3.96 ± 0.01

**Table 5:** The effect of different nitrogen sources on riboflavin production by *G.oxydans* FBFS97.

Nitrogen sources	Riboflavin production mg/L
Tryptone	5.173 ± 0.025
Yeast extract	4.867 ± 0.076
Peptone	2.915 ± 0.210
Malt extract	1.713 ± 0.017

**Figure 5:**
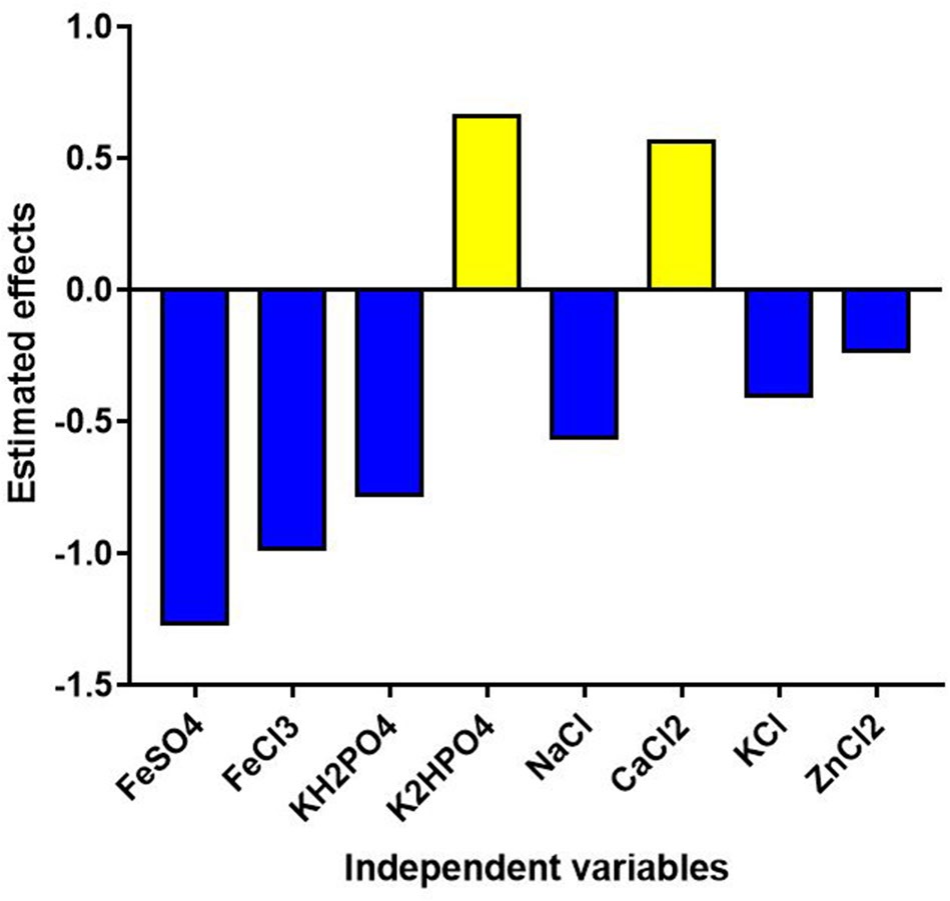
The main effects of the variables affecting riboflavin production by *G.oxydans* FBFS97.

**Figure 6:**
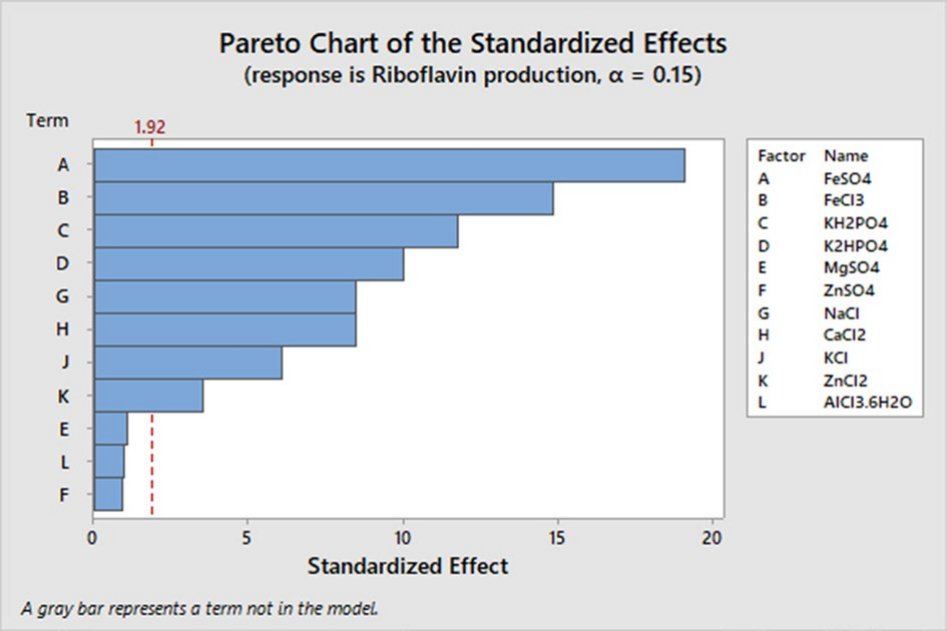
Pareto Chart shows the effect of each variables on riboflavin production by *G.oxydans* FBFS97.

Plackett–Burman design was used to screen and detect the effect of the 11 minerals (FeSO_4_, FeCl_3_, KH_2_PO_4_, K_2_HPO_4_, MgSO_4_, ZnSO_4_, NaCl, CaCl_2_, KCl, ZnCl_2_, and AlCl_3_.6H_2_O) on the riboflavin production using the improved medium. PB experimental design exhibited an obvious variation of riboflavin production from 0.32 to 3.72 mg/l. The maximum riboflavin production (3.72 mg/L) was observed in the run 9, whereas the minimum riboflavin production (0.32 mg/L) was found to be in the run 1(Table 6). Using the multiple regression model revealed that, MgSO_4_, AlCl_3_.6H_2_O, and ZnSO_4_ were insignificant variables with zero effect and zero percent of the contribution, which indicates a higher *P*-value. To avoid the effect of MgSO_4_, AlCl_3_.6H_2_O, and ZnSO_4_ the stepwise regression at alpha 0.15 was applied. Standardized Pareto chart of the PB design exhibited that, the arrangement of significance of the variables effecting on the riboflavin production where the length of each bar in this chart is proportional to the absolute value of its associated regression coefficient or estimated effect. In this study, Pareto chart showed that FeSO_4_, FeCl_3_, and KH_2_PO_4_ were the most effective significant variables on riboflavin production but the effect of these three minerals were all nigative. Figures 5, 6 show the main effects plot of each variable on riboflavin production, we can notice that two variables from the eleven different independent variables called K_2_HPO_4_ and CaCl_2_ affect positively riboflavin production, where the six variables called FeSO_4_, FeCl_3_, KH_2_PO_4_, NaCl, KCl, and ZnCl_2_ affect negatively riboflavin production. Therefore, only the significant variables with positive effect were used for further optimization experiments. The experimental results was obtained by using the following equation:

3 $$\begin{aligned} Y_{{\left( {{\text{riboflavin}}\;{\text{ production}}} \right)}} & = { 1}.{2992 } - 0.{6325} {\text{X}}_{{1}} - 0.{49}0{8} {\text{X}}_{{2}} - 0.{3892} {\text{X}}_{{3}} + 0.{33}0{8} {\text{X}}_{{4}} \\ & \quad - 0.{28}0{8} {\text{X}}_{{7}} + 0.{28}0{8} {\text{X}}_{{8}} - 0.{2}00{8} {\text{X}}_{{9}} - 0.{1175} {\text{X}}_{{{1}0}} \\ \end{aligned}$$

where Y is the response (riboflavin production) and X1, X2, X3, X4, X7, X8, X9, X10 are FeSO_4_, FeCl_3_, KH_2_PO_4_, K_2_HPO_4_,NaCl, CaCl_2_, KCl, and ZnCl_2_ .

**Table 6:** Screened variables (g/L) and their two levels in the PBD, along with riboflavin production. FeSO_4_ (X_1_), FeCl_3_ (X_2_), KH_2_PO_4_ (X_3_), K_2_HPO_4_ (X_4_), MgSO_4_ (X_5_), ZnSO_4_ (X_6_), NaCl (X_7_), CaCl_2_ (X_8_), KCl (X_9_), ZnCl_2_ (X_10_), and AlCl_3_.6H_2_O (X_11_).

Run	Design Matrix											Riboflavin(mg/L)
	X1	X2	X3	X4	X5	X6	X7	X8	X9	X10	X11	
1	+	−	+	−	−	−	+	+	+	−	+	0.32
2	+	+	−	+	−	−	−	+	+	+	−	1.04
3	−	+	+	−	+	−	−	−	+	+	+	0.44
4	+	−	+	+	−	+	−	−	−	+	+	1.21
5	+	+	−	+	+	−	+	−	−	−	+	0.69
6	+	+	+	−	+	+	−	+	−	−	−	0.37
7	−	+	+	+	−	+	+	−	+	−	−	0.70
8	−	−	+	+	+	−	+	+	−	+	−	2.42
9	−	−	−	+	+	+	−	+	+	−	+	3.72
10	+	−	−	−	+	+	+	−	+	+	−	0.37
11	−	+	−	−	−	+	+	+	−	+	+	1.61
12	−	−	−	−	−	−	−	−	−	−	−	2.70

Level of variables	Components (g/L)											
	FeSO_4_	FeCl_3_	KH_2_PO_4_	K_2_HPO_4_	MgSO_4_	ZnSO_4_	NaCl	CaCl_2_	KCl	ZnCl_2_	AlCl_3_.6H_2_O	
Low ( −)	0	0	0	0	0	0	0	0	0	0	0	
High ( +)	0.02	0.02	3	3	1.5	0.02	2.5	0.02	2	0.02	0.005

### Optimization of significant variables by CCD

From the Placket-Burman results, the most effective variables that significantly positive impact riboflavin production by FBFS97 were K_2_HPO_4_ and CaCl_2_, consequently, they were selected for further optimization utilizing central composite design. K_2_HPO_4_ renders as an important phosphorus source for substance vitality such as cell growth and product formation . Also, K_2_HPO_4_ along with KH_2_PO_4_ uses as a pH buffer for the medium^39^.Adding CaCl_2_ to the medium can improve the metabolic activity of bacterial strain. Calcium has an important role on the growth of microorganisms because of its ability to control the cell permeability^40^. In the present study, the CCD was employed to investigate the interactions between the significant variables and determine their optimum values for riboflavin production. A total of 30 experimental combinations for four significant variables (fructose, tryptone, K_2_HPO_4_, and CaCl_2_) were achieved at five levels with the center points replicated six times in the experiment (run order: 4, 18, 20, 22, 23, 29). The range of variables concentrations at different coded levels shown in Table 7. The maximum riboflavin production 23.67 mg/L was observed in run number 16, while the minimum riboflavin production 0.095 mg/L was achieved in run number 28. The design matrix and the experimental and predicted values for riboflavin production noted in Table 7.

**Table 7:** Design matrix of experimental runs for riboflavin production by *G.oxydans* FBFS97 using central composite design (CCD), representing the riboflavin production as affected by fructose (X1), tryptone (X2), K_2_HPO_4_ (X3), and CaCl_2_ (X4), together with the predicted riboflavin production and the levels of variables.

Run	Variables				Riboflavin production mg/L	
	X1	X2	X3	X4	Experimental value	Predicted value
1	− 2	0	0	0	12.21	11.63
2	− 1	− 1	1	− 1	10.66	9.43
3	0	0	2	0	1.75	4.29
4	0	0	0	0	18	16.63
5	1	1	− 1	1	11.93	12.58
6	0	0	− 2	0	9.03	7.40
7	0	0	0	− 2	19.35	18.62
8	1	1	− 1	− 1	12.03	10.80
9	1	− 1	1	1	0.83	− 1.25
10	1	− 1	− 1	− 1	13.14	13.06
11	− 1	1	− 1	− 1	10.28	11.76
12	− 1	− 1	− 1	1	8.46	5.88
13	− 1	1	− 1	1	10.98	12.10
14	− 1	− 1	− 1	− 1	8.25	10.56
15	0	− 2	0	0	1.21	2.48
16	− 1	1	1	1	23.67	23.16
17	0	2	0	0	15.91	15.54
18	0	0	0	0	16.9	16.63
19	1	1	1	− 1	3.72	5.72
20	0	0	0	0	18	16.63
21	0	0	0	2	17.13	18.81
22	0	0	0	0	15.7	16.63
23	0	0	0	0	14.4	16.63
24	− 1	− 1	1	1	6.86	7.85
25	1	− 1	− 1	1	9.01	9.82
26	1	− 1	1	− 1	0.29	− 1.11
27	1	1	1	1	13.13	10.59
28	2	0	0	0	0.095	1.57
29	0	0	0	0	16.8	16.63
30	− 1	1	1	− 1	20.82	19.73

Levels	Fructose (g/L)	Tryptone (g/L)	K_2_HPO_4_ (g/L)	CaCl_2_ (g/L)		
− 2	25.00	2.5	1.00	0.02		
− 1	50.00	5.00	3.00	0.03		
0	75.00	7.00	5.00	0.04		
1	100.00	10.00	7.00	0.05		
2	125.00	12.5	9.00	0.06		

Design Expert 12 software was used to analyze the experimental data, and the resultant second-order polynomial equation for riboflavin production was as the following:

4 $$\begin{aligned} Y_{{\left( {{\text{riboflavin}}\;{\text{production}}} \right)}} & = { 16}.{63 } - { 2}.{\text{51X}}_{{1}} + { 3}.{\text{26X}}_{{2}} - \, 0.{\text{7775X}}_{{3}} + \, 0.0{\text{475X4 }}{-} \, 0.{\text{8663X}}_{{1}} {\text{X}}_{{2}} - { 3}.{\text{26X}}_{{1}} {\text{X}}_{{3}} \\ & \quad + \, 0.{\text{36X}}_{{1}} {\text{X}}_{{4}} + { 2}.{\text{27X}}_{{2}} {\text{X}}_{{3}} + { 1}.{\text{25X}}_{{2}} {\text{X}}_{{4}} + \, 0.{\text{7738X}}_{{3}} {\text{X}}_{{4}} {-}{ 2}.{\text{51X}}_{{1}}^{{2}} {-}{ 1}.{\text{91 X}}_{{2}}^{{2}} - { 2}.{7}0{\text{ X}}_{{3}}^{{2}} + \, 0.{52}0{\text{5 X}}_{{4}}^{{2}} \\ \end{aligned}$$

where Y is the predicted value of riboflavin production, X_1_ is fructose, X_2_ is tryptone, X_3_ is K_2_HPO_4_, and X_4_ is CaCl_2_.

### Statistical analysis

The statistical significance of the fitted model was evaluated by multiple regression and the analysis of variance (ANOVA) which was tested using Fisher’s test value Table 8. The model *F*-value of 18.80 with *P* < 0.0001 implies that the model is highly significant and there was only a 0.01% chance that this high F-value could occur due to noise. The lack of fit was not significant relative to the pure error (*P* ˃0.05). Furthermore, it can be noticed from the degree of significant that X_1_, X_2_, X_1_X_3_, X_2_X_3_, X_2_X_4_, X_1_^2^, X_2_^2^ and X_3_^2^ are significant model terms. The coefficient of determination (R^2^) of the model was 0.9461 which indicated that the model could be used reliably for the riboflavin production in this study. The Predicted R^2^ of 0.7240 is in reasonable agreement with the Adjusted R^2^ of 0.8957 which implied a good adjustment between the predicted and observed values. Adeq Precision ratio of 16.5602 indicates an adequate signal to noise ratio. The positive coefficient values indicate that individual effect (X_2_, X_4_), interaction effects (X_1_X_4_, X_2_X_3_, X_2_X_4,_ X_3_X_4_) and quadratic effect (X_4_^2^) increase the production of riboflavin, whereas other negative coefficient values indicate to decrease in riboflavin production.

**Table 8:** Results of the analysis of variance (ANOVA) for the quadratic model used for optimizing riboflavin production by *G.oxydans* FBFS97.

Source	Coefficient	SS	df	MS	*F*-value	*P* value	Significance level
Model		1,143.71	14	81.69	18.80	< 0.0001	Significant
X_1_	− 2.51	151.76	1	151.76	34.92	< 0.0001	Significant
X_2_	3.26	255.71	1	255.71	58.83	< 0.0001	Significant
X_3_	− 0.7775	14.51	1	14.51	3.34	0.0877	N
X_4_	0.0475	0.0541	1	0.0541	0.0125	0.9126	N
AB	− 0.8663	12.01	1	12.01	2.76	0.1173	N
AC	− 3.26	170.17	1	170.17	39.15	< 0.0001	Significant
AD	0.3600	2.07	1	2.07	0.4771	0.5003	N
BC	2.27	82.63	1	82.63	19.01	0.0006	Significant
BD	1.25	25.15	1	25.15	5.79	0.0295	Significant
CD	0.7738	9.58	1	9.58	2.20	0.1584	N
A^2^	− 2.51	172.47	1	172.47	39.68	< 0.0001	Significant
B^2^	− 1.91	99.62	1	99.62	22.92	0.0002	Significant
C^2^	− 2.70	199.69	1	199.69	45.94	< 0.0001	Significant
D^2^	0.5205	7.43	1	7.43	1.71	0.2107	N
Residual		65.20	15	4.35			
Lack of fit		55.50	10	5.55	2.86	0.1286	Not significant
Pure error		9.69	5	1.94			
Cor total		1,208.90	29				
Std.Dev	2.08		R^2^		0.9461		
Mean	11.36		Adjusted R^2^ 0.8957				
C.V.%	18.35		Predicted R^2^		0.7240		
Press	333.65		Adeq Precision		16.5602		

*N* non-significant, *df* degree of freedom, *C.V* coefficient of variation, *P* level of significant, *F* Fisher’s function.

### Response surface plots

 The three-dimensional response surface curves were plotted to explain the interaction of the variables and obtain the optimal level of each variable required for riboflavin production by FBFS97. 3D surface plots were created for the response (riboflavin production) at any two independent variables while keeping the other variables at their 0 level.

**Figure 7:**
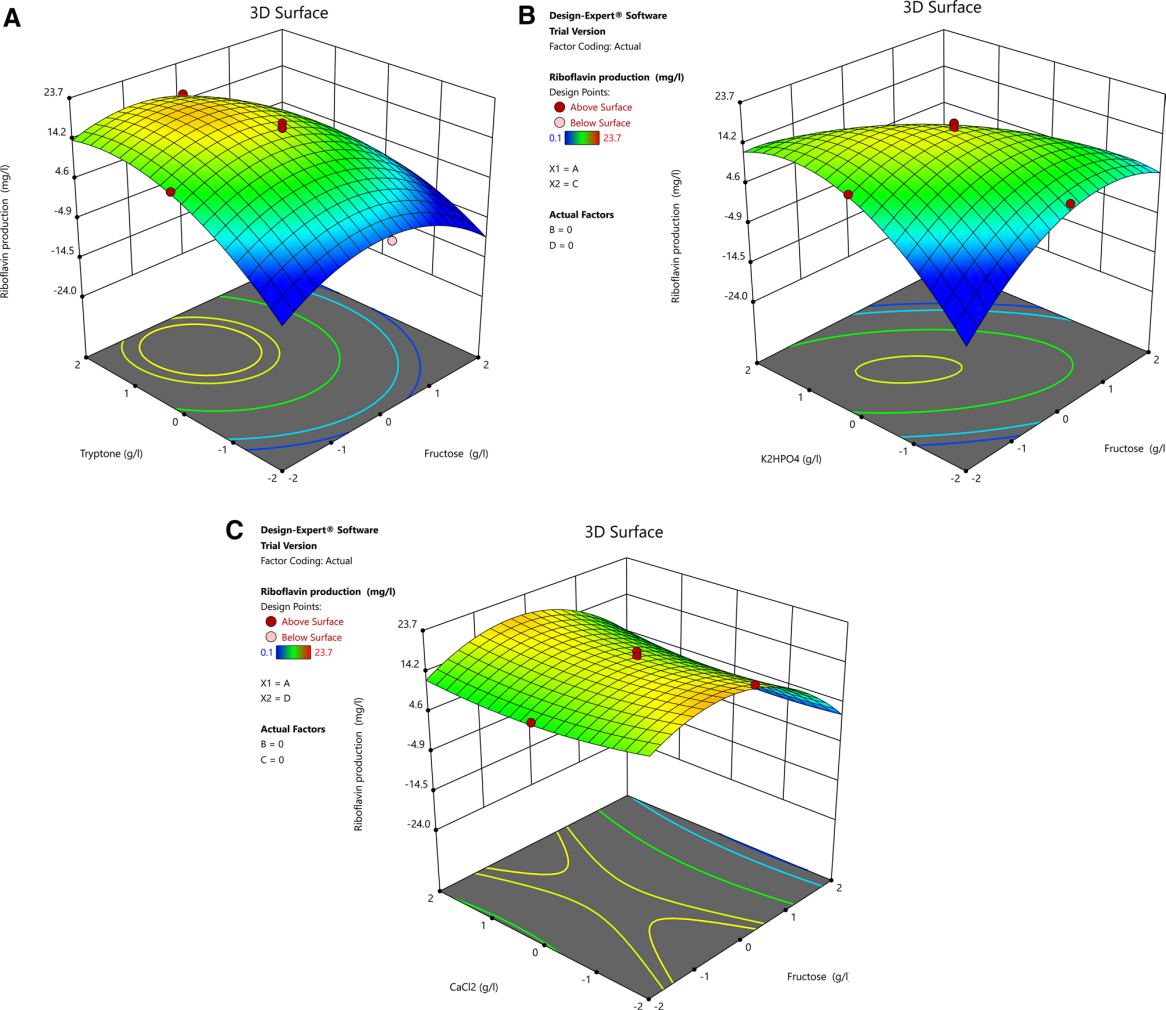

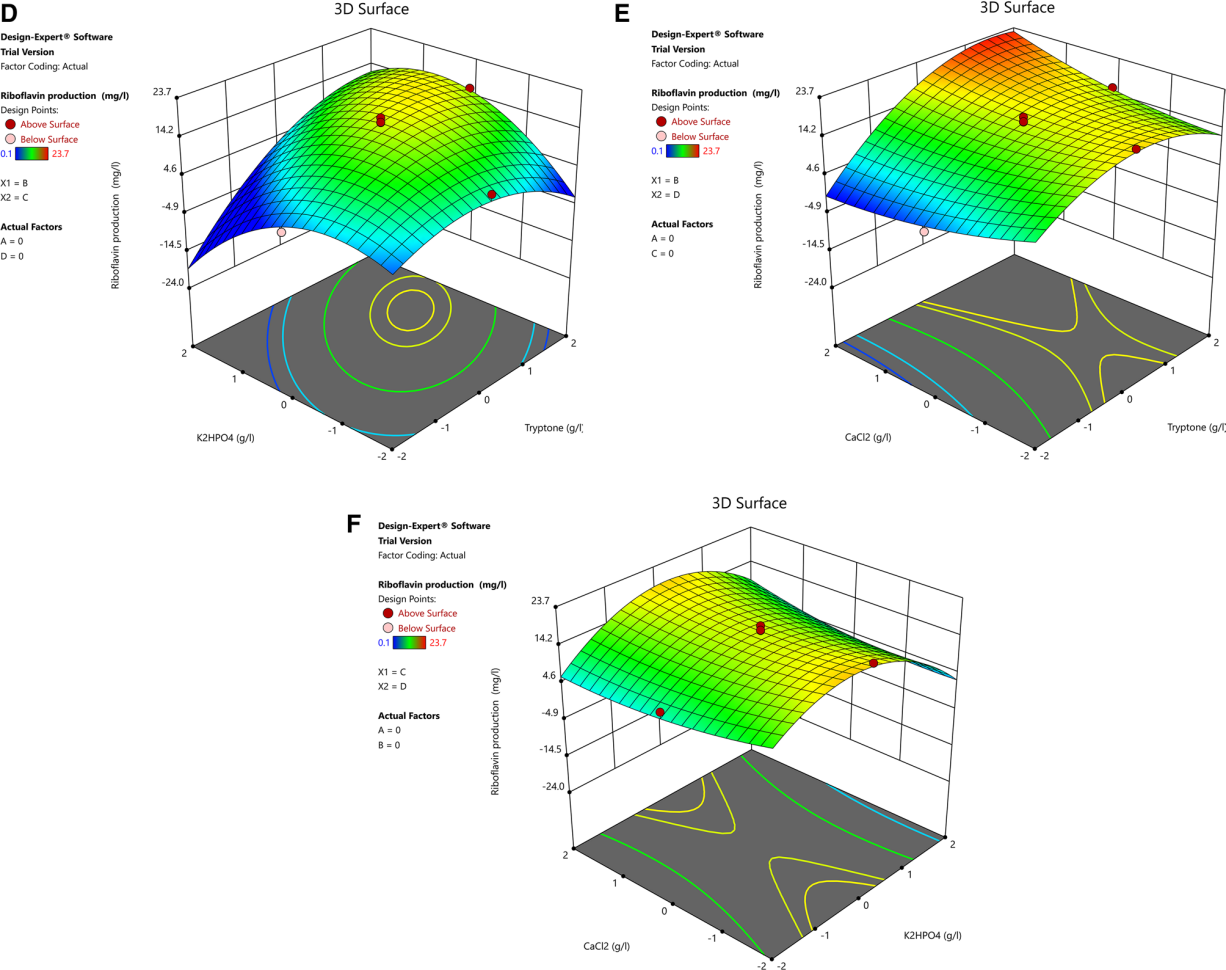
Three-dimensional RSM plots for riboflavin production showing the effect of concentration of fructose, tryptone, K2HPO4 and CaCl2 and their mutual interaction in pairs when the other two variables were constant at zero level. Interactions of (A) fructose and tryptone, (B) fructose and K2HPO4, (C) fructose and CaCl2, (D) tryptone and K2HPO4, (E) tryptone and CaCl2, and (F) K2HPO4 and CaCl2.

Figure 7A shows the interaction effect of fructose and tryptone concentrations in riboflavin production. As can be seen in the plot, the increase of fructose concentration leads to a decrease in riboflavin production. While the increase of tryptone concentration enhanced the riboflavin production until the center point and then riboflavin production decreased gradually with a further increase in the concentration of this variable. The interaction effect between these two variables were not significant, pointing that out there is no significant correlation between them, thus they did not help much in the riboflavin production increasing. Figure 7B reveals the effect of fructose and K_2_HPO_4_ concentrations on riboflavin production.Where a negative effect on riboflavin production was observed at the higher concentrations of both variables. This result was in agreement with the finding of Marjan et al^41^. who used fructose as the only carbon source along with K_2_HPO_4_ to enhance the production of riboflavin by *Bacillus subtilis* ATCC 6,051, also they mentioned to that the excessive fructose and K_2_HPO_4_ in the culture medium can be effected negatively on riboflavin production. The plot of Fig. 7C indicates the effect of the interaction of fructose and CaCl_2_ on riboflavin production. The maximum production of riboflavin was obtained at the lowest level of CaCl_2_ and level 0 of fructose and then riboflavin production was decreased from the central point along with further concentrations increase of both variables. Figure 7D represents the effect of tryptone and K_2_HPO_4_ on the production of riboflavin by FBFS97. A gradual increase of riboflavin production was found when the initial tryptone and K_2_HPO_4_ concentrations were increased until the optimum value and then riboflavin production decreased with further increase in the concentration of both variables. Figure 7E depicts the interaction of tryptone and CaCl_2_. Riboflavin production increased as tryptone and CaCl_2_ concentrations increased. The optimum riboflavin production was observed at the central point and then a slight decrease was observed with further increase in the concentration of both variables. Figure 7F indicates the effect of K_2_HPO_4_ and CaCl_2_ on riboflavin production in the FBFS97 fermentation medium. At a moderate level of K_2_HPO_4_ the production riboflavin was high, as the CaCl_2_ was at its low level. A slight decrease was noticed towards the central point as the concentration of both variables at their middle level, then a decline in the curve was observed with further increase of K_2_HPO_4_. In addition, the interaction terms between these variables were not significant, indicating that there is no significant correlation between the two variables, thus the interaction between them did not help much in increasing the production of riboflavin.

### Validation of the model results

In order to confirm the reliability of the statistical design and validate the results, an experiment with the new medium composition which predicted by the statistical model was carried out. It was obtained that the optimal levels of the variables for riboflavin production by *G.oxydans* FBFS97 were fructose 25 g/L, tryptone 12.5 g/L, K_2_HPO_4_ 9 g/L, and CaCl_2_ 0.06 g/L. The production of riboflavin by FBFS97 was 23.24 mg/L found from the validation experiment was close to the predicted value of 23.2 mg/L, which confirms the accuracy of the model with a great degree of desirability of 97.7% . Compared with some strains of lactic acid bacteria, the production of riboflavin by FBFS97 as a wild strain was higher than the riboflavin production by *Lactobacillus fermentum* GKJFE (3.49 mg/L), *Lactobacillus plantarum* NCDO1752 (0.6 mg/L), and *Leuconostoc lactis* NZ9000 (0.7 mg/L) as mutant strains^42^.

## Conclusion

A new riboflavin producer *G.oxydans* FBFS97 was isolated from the soil sample collected in Wuhan, China. Then, the effect of medium composition on riboflavin production by FBFS97 was investigated. Fructose and tryptone were chosen as suitable sources of carbon and nitrogen. PBD was applied to choose the most significant minerals effective on riboflavin production by FBFS97. The results of CCD coupled with response surface modeling (RSM) displayed that the optimum concentrations of the selected variables were fructose 25 g/L, tryptone 12.5 g/L, K_2_HPO_4_ 9 g/L, and CaCl_2_ 0.06 g/L with the maximum riboflavin production 23.24 mg/L. The results of this study revealed that *G.oxydans* FBFS97 could be a new potential candidate for the industrial application of riboflavin production.
